# The Role of Type 2 Diabetes for the Development of Pathogen-Associated Cancers in the Face of the HIV/AIDS Epidemic

**DOI:** 10.3389/fmicb.2017.02368

**Published:** 2017-11-29

**Authors:** Melissa J. Blumenthal, Sylvia Ujma, Arieh A. Katz, Georgia Schäfer

**Affiliations:** Receptor Biology Research Unit, Division of Medical Biochemistry and Structural Biology, Institute of Infectious Disease and Molecular Medicine, Department of Integrative Biomedical Sciences, Faculty of Health Sciences, SA-MRC Gynecology Cancer Research Centre, University of Cape Town, Cape Town, South Africa

**Keywords:** type 2 diabetes, pathogen-associated cancers, HIV/AIDS, HPV, KSHV, low- and middle-income countries, sub-Saharan Africa

## Abstract

The contribution of HIV to the development of pathogen-associated cancers has long been recognized, as has the contribution of type 2 diabetes for the development of several types of cancer. While HIV/AIDS-associated immunosuppression reduces immunosurveillance and indirectly contributes favorably to cancerogenesis, diabetes directly increases cancer development due to chronic low-grade inflammation, dysregulated glucose metabolism, hyperactivation of insulin-responsive pathways, and anti-apoptotic signaling. Pathogen-associated cancers contribute significantly to the cancer burden particularly in low- and middle-income countries. In those countries, the incidence of type 2 diabetes has increased alarmingly over the last decades, in part due to rapid changes in diet, lifestyle, and urbanization. It is likely that the HIV/AIDS epidemic and the steadily increasing rate of type 2 diabetes display synergistic effects on oncogenesis. Although this possible link has not been extensively investigated, it might become more important in the years to come not least due to the stimulating effects of antiretroviral therapy on the development of type 2 diabetes. This review provides an overview of the current understanding of pathogen- and diabetes- associated cancers with focus on geographical regions additionally burdened by the HIV/AIDS epidemic. As both HIV and carcinogenic infections as well as the onset of type 2 diabetes involve environmental factors that can be avoided to a certain extent, this review will support the hypothesis that certain malignancies are potentially preventable. Deploying effective infection control strategies together with educational policies on diet and lifestyle may in the long term reduce the burden of preventable cancers which is of particular relevance in low-resource settings.

## Introduction

The development of cancer is a complex multistep process. It is well-understood that the lack of responsiveness to signals from the microenvironment together with the accumulation of aberrations in multiple cellular regulatory systems can eventually lead to the characteristic loss of growth control displayed by cancer cells (Hanahan and Weinberg, 2011). Oncogenesis is typically initiated by a non-lethal genetic or epigenetic alteration leading to abnormal proliferation of a single cell. Apart from the genetic constitution of an individual, this can be caused by multiple agents including radiation, chemicals and persistent infection with oncogenic microbes, the latter causing about 15–20% of all human cancers (Plummer et al., 2016).

Although necessary, initiation alone is not sufficient for tumor formation. In addition, tumor promoters are needed to induce the initiated cells to transform and become malignant. Tumor promoters are generally non-tumorigenic by themselves but rather reversibly stimulate cell proliferation and/or provide a tumor-friendly environment (e.g., hormones, chronic inflammation, and immunosuppression). The combined action of initiators and promoters can then eventually give rise to cells that become gradually and irreversibly malignant through a progressive series of alterations (Vincent and Gatenby, 2008).

Smoking and alcohol consumption, obesity and physical inactivity are the worldwide leading risk factors for cancer death, with oncogenic pathogens significantly contributing to cancer incidence in low-and middle-income countries which are often additionally burdened by the HIV/AIDS epidemic (Danaei et al., 2005). The leading types of cancer-causing infections worldwide are *Helicobacter pylori*, human papillomavirus (HPV), hepatitis B virus (HBV) and hepatitis C virus (HCV), while Kaposi’s sarcoma-associated herpesvirus (KSHV) is particularly important in the sub-Saharan African context. Co-infection with human immunodeficiency virus (HIV) substantially enhances the incidence of pathogen-associated cancers (Working Group on the Evaluation of Carcinogenic Risks to Humans [IARC], 2012). Although HIV is not considered an oncogenic virus, it indirectly impacts on cancerogenesis through immune deficiency and impaired immune surveillance, thereby increasing the effects of oncogenic infections (Working Group on the Evaluation of Carcinogenic Risks to Humans [IARC], 2012). Cancer-promoting effects have also been ascribed to the chronic metabolic and hormonal disturbances as seen in type 2 diabetes (Giovannucci et al., 2010), a condition that is no longer considered a disease primarily affecting the industrialized world but has become increasingly common in low-resource settings (Hu, 2011).

All three conditions, namely type 2 diabetes, HIV-associated immunosuppression and oncogenic pathogen infections, not only contribute to cancer development, they are also largely preventable. Fundamental changes in public policies with regard to diet and lifestyle modifications, education and training on infection prevention as well as nation-wide prophylactic vaccination programs against HBV and HPV are predicted to have a significant impact on the cancer burden in low- and middle- income countries (Sylla and Wild, 2012).

## Pathogen-Associated Cancer Development in the Context of HIV/AIDS

Cancers attributable to infectious agents are an important component of the global cancer burden. Assessments dating from 1990 to 2012 have attributed 1/6th of global cancer cases to infectious etiologies and in sub-Saharan Africa, which is heavily burdened with HIV/AIDS, this is greater than 30% (Pisani et al., 1997; Parkin, 2006; de Martel et al., 2012; Plummer et al., 2016). Oncogenic infectious agents include viruses: HCV, HBV, high-risk HPV, KSHV, Epstein–Barr virus (EBV), human T-cell lymphotrophic virus type-1 (HTLV-1) and Merkel cell polyomavirus (MCPyV) (Houben et al., 2010; Working Group on the Evaluation of Carcinogenic Risks to Humans [IARC], 2012; Schäfer et al., 2015); bacteria: *Helicobacter pylori* (Forman et al., 1991; Nomura et al., 1991; Parsonnet et al., 1991); and parasites: *Opisthorchis viverrini*, *Clonorchis sinensis*, and *Schistosoma haematobium* (International Agency for Research on Cancer Working Group, 1994; Honjo et al., 2005; Bouvard et al., 2009). Additionally, HIV is classified as carcinogenic, although its mechanism is indirect via cell-mediated immune deficiency and needs to be in conjunction with another infectious agent (Working Group on the Evaluation of Carcinogenic Risks to Humans [IARC], 2012). Not surprisingly, the exceptional elevation of pathogen-associated cancers in the developing world is, not least, exacerbated in the high HIV/AIDS context (Parkin et al., 2005; Grulich et al., 2007).

The introduction of the highly active antiretroviral therapy (HAART) strategy has substantially reduced the number of AIDS-related deaths and extended the lifespans of HIV infected individuals (Palmisano and Vella, 2011). This extended lifespan has led to the emergence of a range of HIV-associated malignancies, again particularly burdening sub-Saharan Africa, that were not often seen preceding the introduction of HAART when average lifespan following HIV infection was significantly less (Frisch et al., 2001; Grulich et al., 2007; Coghill et al., 2013; Antiretroviral Therapy Cohort Collaboration, 2017). In contrast, HAART also had a profound preventative effect on some, but not all, HIV-associated malignancies. Kaposi’s sarcoma (KS) incidence in Western countries was reported to decrease by greater than 90% from 1994 to 2003, spanning the introduction of HAART in 1996 (Mocroft et al., 2004). Similarly, incidence of EBV-associated non-Hodgkin lymphoma decreased by greater than 40% after the introduction of HAART in Western countries (International Collaboration on HIV and Cancer, 2000). However, incidences of HPV-associated cervical cancer and non-AIDS defining cancers have not yet been seen to be reduced (International Collaboration on HIV and Cancer, 2000). Although promising, the reductions of incidences of particularly KS and non-Hodgkin lymphoma in Western countries, has not been mirrored in resource-limited, developing countries. In fact, GLOBOCAN age standardized incidence rates reported in 2002 and 2012, indicate an increase in incidence of non-Hodgkin lymphoma in males and females in Southern and Northern Africa (Parkin et al., 2005; Torre et al., 2015). This is corroborated in a Ugandan study over the period 1991–1995 to 2002–2006, which reports an annual increase in incidence of non-Hodgkin lymphoma by 6.7 and 11.0% in men and women, respectively (Parkin et al., 2010). Similarly, KS incidence in Ugandan women has increased (1.4% annually, 10% over the period), while in men incidence has slightly decreased (2.8% annually, 30% over the period); this in contrast to the large (>90%) reductions in KS incidence in Western countries over a similar period, and concomitant with the large scale roll-out of HAART (Mocroft et al., 2004; Parkin et al., 2010; Casper, 2011). This disparity has been attributed to a delayed and stilted availability of HAART in low-resource settings; indeed, it has been noted that even in sub-Saharan countries with relatively well-established antiretroviral therapy (ART) programs, KS incidence has not decreased as expected (Casper, 2011). Further, Nguyen et al. (2008) found that up to half of AIDS-related KS patients treated with HAART and chemotherapy never achieved total remission, which was in concordance with other studies (Dupin et al., 1999; Dupont et al., 2000; Bihl et al., 2007; Nguyen et al., 2008). It is therefore predicted that cancers caused by infectious agents will become a burgeoning complication of long-term HIV infection (Grulich et al., 2007; Sasco et al., 2010; Casper, 2011).

Oncogenic organisms infect, but do not kill their target cells, leading to persistent infection. This is facilitated by various immune evasion strategies driven by the expression of pathogen-encoded proteins and subversion of cellular regulation of proliferation and apoptosis (McLaughlin-Drubin and Munger, 2008; Mesri et al., 2014). Perhaps the best example of this is oncogenic HPV infection of basal cells of the cervical epithelium, which precedes development of virtually all cases of cervical cancer by decades (Woodman et al., 2007; Castellsague, 2008). HPV oncogenes E6 and E7 mitigate host innate immune responses through inhibition of the interferon pathway (Ronco et al., 1998; Park et al., 2000) and similarly, E5 oncoprotein causes downregulation of MHC class I (Campo et al., 2010). Likewise, HBV and HCV infection, which together account for approximately 80% of hepatocellular carcinoma cases, promote cirrhosis through non-cytocidal chronic infection of hepatocytes (Parkin et al., 2005; El-Serag, 2012). HBV-encoded X antigen (HBx) and HCV-encoded core, non-structural protein 5A (NS5A) and NS3 antigens inhibit innate antiviral signaling pathways as well as apoptosis, conferring a survival advantage to infected hepatocytes (Mesri et al., 2014). Persistent infection through immune evasion is further facilitated in an immunosuppressive environment, such as in HIV infection, where host-mediated anti-tumor responses are extinguished.

Persistent infection can be accompanied by chronic inflammation. Persistent HBV and HCV infection lead to chronic inflammation of the liver (hepatitis) which promotes cancer development (Mesri et al., 2014). Similarly, key to the oncogenic potential of *Helicobacter pylori*, to which approximately 90% of new stomach cancer cases are attributed, is its ability to persist in the gastric mucosa for decades, eliciting chronic inflammation through the expression of virulence factors *CagA* (cytotoxin-associated gene A) and *VacA* (vacuolating cytotoxin A) (Wang et al., 2014; Plummer et al., 2015, 2016). Chronic low-grade inflammation is also present in HIV infected individuals on ART treatment and is associated with cancer development (Marks et al., 2013; Maartens et al., 2014).

Persistent oncogenic infection is necessary but often not sufficient to trigger carcinogenesis, but rather requires precipitating co-factors for the development of malignancy (McLaughlin-Drubin and Munger, 2008; Mesri et al., 2010). Often, immunosuppression plays this role, made evident by the enhanced incidence of infection-related cancers and cancers suspected to have infectious etiology seen in both HIV-positive patients and immunosuppressed post-transplant cohorts (Grulich et al., 2007). Of those, KS is the most common AIDS-related malignancy and a significant public health burden in sub-Saharan Africa, where KSHV seroprevalence rates as high as 50% have been reported (Mesri et al., 2010). While KSHV infection accounts for 100% of KS cases, alone it is asymptomatic and not sufficient for tumorigenesis, requiring HIV-related or another form of immunosuppression [e.g., standardized incidence ratio (SIR) for KS in HIV/AIDS cohort versus transplant cohort is 3640.0 (3326–3976) versus 208.0 (114–349), respectively] amongst other potential co-factors, to trigger KS development (Grulich et al., 2007; Mesri et al., 2010; Plummer et al., 2016). Moreover, due to shared routes of transmission, HPV and HIV coinfection is common and HIV infection increases the probability of HPV persistent infection resulting in increased relative risk [5.8 (3.0–11.3)] of cervical cancer development compared to HPV infection in the absence of HIV (Strickler et al., 2005; Grulich et al., 2007; Working Group on the Evaluation of Carcinogenic Risks to Humans [IARC], 2012). Similarly, HIV co-infection with either HBV or HCV increases the rate of progression of HBV- or HCV-mediated liver damage and the SIR of HBV/HCV-mediated liver cancer [5.22 (3.32–8.20)] (Graham et al., 2001; Grulich et al., 2007; Chen et al., 2009; Nikolopoulos et al., 2009).

Furthermore, a number of infectious cancers show distinct geographical distributions or population-specific prevalence. Before the HIV epidemic, KS was considered rare except for hotspots in the Mediterranean and Eastern Europe (Classic KS) and Central and Eastern Africa (Endemic KS). Now, AIDS-related KS is prevalent across sub-Saharan Africa, but its peculiar geographical epidemiology has led to speculation that host genetic factors may influence seroconversion after exposure to KSHV and/or subsequent KS development (Mesri et al., 2010; Cavallin et al., 2014). Interestingly, while HCV is an important risk factor for the development of hepatocellular carcinoma in the United States, Europe and Japan, HBV contributes more substantially to the development of liver cancer globally and particularly in countries with low human development index (Barth et al., 2010; Chung et al., 2010; Plummer et al., 2016).

Besides HIV immunosuppression as outlined above, environmental factors such as host diet, physical inactivity, behaviors such as smoking, reproductive factors, and co-factors leading to smoldering chronic inflammation are thought to further influence the natural history of oncogenic infections and as a consequence of globalization, these are thought to become more important in the developing world (Parkin et al., 2005; Ajuwon et al., 2009; Plummer et al., 2016).

## Diabetes-Associated Cancer Development in the Context of HIV/AIDS

Type 2 diabetes is a non-communicable disease, characterized by insulin resistance and hyperglycemia (Zaccardi et al., 2016).

Globally, type 2 diabetes is a major problem, but prevalence of the disease is particularly rapidly increasing in low- and middle-income countries (Guariguata et al., 2014). This is largely due to the changes in lifestyle and diet brought about by rapid urbanization, such as decreased physical activity as well as increased consumption of refined carbohydrates and sugar sweetened beverages (Hu, 2011). On that note, it was shown that only 1–2 servings of sugar-sweetened beverages per day increase diabetes risk by 26% (Malik et al., 2010). In 2013, low- and middle-income countries had the highest prevalence of type 2 diabetes and it is projected that Africa will experience a 109% increase in cases over the next 22 years (Guariguata et al., 2014). The most undiagnosed cases of type 2 diabetes occur in low- and middle-income countries due to a lack of resources necessary for diagnosis, meaning that often the disease is only detected after complications have already developed (Beagley et al., 2014). The Lancet Diabetes & Endocrinology Commission on diabetes in sub-Saharan Africa provides a comprehensive and up-to-date analysis of the vast economic burden that diabetes places on the resource-constrained health systems found in those regions (Atun et al., 2017).

As outlined above, low- and middle-income countries are also highly burdened by the HIV/AIDS epidemic, and 2–4 fold higher prevalence of dysglycemia in HIV-infected individuals in South Africa has been reported (Dave et al., 2011; Levitt et al., 2016). Although the mechanisms are not well-understood, the presence of HIV infection itself may contribute to diabetogenesis, both directly through inflammation and immune activation, and indirectly through immunodeficiency (Kalra and Agrawal, 2013; Lake and Currier, 2013; Levitt et al., 2016). Moreover, treatment by HAART may be directly or indirectly linked to the development of type 2 diabetes in HIV-infected patients (Hadigan and Kattakuzhy, 2014). The increased life expectancy of HIV-infected patients on HAART treatment has dramatically changed the natural history of HIV infection (Samaras, 2009), resulting in the emergence of more chronic illnesses, including type 2 diabetes (Hasse et al., 2011). Moreover, certain nucleoside reverse transcriptase inhibitors (NRTIs) and protease inhibitors (PIs) have been associated with the development of insulin resistance and an increased incidence of type 2 diabetes in HIV-infected individuals, though the literature has been contentious about whether PIs do indeed play a role (Behrens et al., 1999; Brown et al., 2005; Tien et al., 2007; De Wit et al., 2008; Capeau et al., 2012). A large cross-sectional study recently published reported a significantly higher odds ratio (OR) of diabetes and metabolic syndrome (OR 3.85 and 1.45, respectively, 95% CI) among ART-exposed patients compared to their naïve counterparts, although the association between ART and diabetes was not interpreted as cause and effect (Nduka et al., 2017). HAART is also known to cause lipodystrophy and hyperlipidemia, conditions common to insulin resistance and type 2 diabetes, therefore indirectly increasing the risk of development of type 2 diabetes in HIV-infected individuals who are on HAART (Samaras, 2009). The high prevalence of HIV/AIDS in today’s era of HAART may therefore further substantially contribute to the increase in type 2 diabetes predicted for low- and middle-income countries.

Of the various complications associated with type 2 diabetes, cancer development, incidence, prognosis, and mortality have been linked to the long-term effects of the disease (Giovannucci et al., 2010). For example, a meta-analysis of 23 studies reported increased mortality across all cancer types in diabetes patients (HR 1.41; 95% CI, 1.28–1.55) (Barone et al., 2008). Specifically, an increased risk of colon cancer has been associated with type 2 diabetes [summary relative risk (SRR) = 1.30] (Larsson et al., 2005), and a cohort study showed that diabetic patients with stages 2 and 3 colon cancer had higher mortality rates and cancer recurrence as compared to non-diabetic patients (Meyerhardt et al., 2003). Individuals with type 2 diabetes had a SRR of 1.94 for the development of pancreatic cancer (Ben et al., 2011) and a SRR of 2.01 for hepatocellular carcinoma development (Wang et al., 2012). Another study described synergistic interactions between diabetes mellitus and hepatocellular carcinoma (OR, 9.9; 95% CI, 2.5–39.3) which was further found to be strikingly associated with heavy alcohol consumption and chronic hepatitis virus infection (OR, 53.9; 95% CI, 7.0–415.7) (Hassan et al., 2002). Type 2 diabetes has also been associated with a 42% increase in kidney cancer risk (Larsson and Wolk, 2011) as well as a 20% increase in risk of developing breast cancer (Larsson et al., 2007). Overall, type 2 diabetes is associated with increased mortality in cancer patients and an overall poor prognosis of cancer (Barone et al., 2008).

Both type 2 diabetes and cancer are highly complex diseases affecting many cellular processes. However, they share several risk factors, such as alcohol consumption, smoking, obesity, diet and physical inactivity, but the possible biological links between the two diseases are not yet completely understood. Possible mechanisms for a direct link between cancer and type 2 diabetes include hyperglycemia, hyperinsulinemia, and inflammation. Firstly, hyperglycemia has been suggested to be one of the possible influences of type 2 diabetes on cancer, mainly due to the Warburg effect: a well-known observation that cancer cells tend to undergo aerobic glycolysis and therefore consume more glucose than normal cells in order to accumulate precursor molecules for biomass rather than energy production (Vander Heiden et al., 2009). Additionally, high levels of glucose are linked to increases in WNT signaling, which enhances proliferation (Garcia-Jimenez et al., 2014), as well as upregulation of the oxidative response genes, leading to increased reactive oxygen species and mutations (Turturro et al., 2007). Secondly, hyperinsulinemia may also be a potential link between type 2 diabetes and cancer, as insulin is not only a metabolic hormone but also a growth factor that has anti-apoptotic and mitogenic effects via activation of the insulin receptor (Vigneri et al., 2016). Of the different insulin receptor isoforms the A isoform, which has predominant mitogenic activity, is often overexpressed in cancer cells, providing a selective growth advantage to malignant cells when exposed to insulin (Vigneri et al., 2016). High levels of circulating insulin due to insulin resistance or insulin treatment also result in reduced insulin-like growth factor binding protein (IGFBP), leading to increased insulin-like growth factor 1 (IGF-1), which has more potent anti-apoptotic and mitogenic effects than insulin (Giovannucci et al., 2010). However, high levels of insulin can also spill over to IGF-1 receptors, and indeed it was shown that both the insulin receptor and the IGF-1 receptor can act as identical portals to the regulation of gene expression with differences between insulin and IGF-1 effects due to a modulation of the amplitude of the signal created by the specific ligand-receptor interaction (Boucher et al., 2010). Hyperinsulinemia also indirectly leads to increased levels of estrogen and androgens by reducing the hepatic synthesis of sex hormone-binding globulin (Calle and Kaaks, 2004); aberrant steroid hormone metabolism is associated with a higher risk of certain cancers, including breast and endometrial cancer. Lastly, excessive caloric intake often associated with urbanization and an increasing Westernized lifestyle leads to obesity which is not only a well-known risk factor for the development of type 2 diabetes but is also linked to cancer development (Hjartaker et al., 2008). In South Africa, 68% of women and 31% of men are overweight or obese (Department of Health, 2016). Additionally, consumption of sugar sweetened beverages has approximately doubled in rural areas since 2005, with 56% of women and 63% of men consuming these beverages, which further contributes to the increasing prevalence of obesity here (Vorster et al., 2014). Chronic low-grade adipose tissue inflammation is not only a symptom of obesity but is also known to be a recognizable feature of the metabolic syndrome and a major cause of the decreased insulin sensitivity seen in type 2 diabetes (van Kruijsdijk et al., 2009; Shu et al., 2012). Moreover, obesity can induce sustained systemic production of reactive oxygen species which can eventually lead to somatic mutations and neoplastic transformation (Manna and Jain, 2015). As an endocrine organ, excess adipose tissue secretes elevated levels of various cytokines, hormones and growth factors including interleukin-6 (IL-6), tumor necrosis factor alpha (TNF-α), plasminogen activator inhibitor-1 (PAI-1) and leptin that can alter the body’s ability to appropriately respond to insulin (van Kruijsdijk et al., 2009). TNF-α has been shown to promote cell survival through the NF-κB pathway, and IL-6 mediates cell proliferation and survival through the JAK/STAT pathway (van Kruijsdijk et al., 2009). Type 2 diabetes is therefore associated with a general dysregulated innate immune response (Paich et al., 2013). Together with metabolic disruptions in insulin signaling, this manifestation of immune dysfunction common to obesity and type 2 diabetes present a favorable environment for cancer cell proliferation, invasion, and survival (Allavena et al., 2008; van Kruijsdijk et al., 2009).

## The Link Between Type 2 Diabetes and Pathogen-Associated Cancers in the Context of HIV/AIDS

Low- and middle-income countries are projected to carry the majority of the world-wide cancer burden over the next decades, highlighting the emergence of cancer as a major public health problem (Sylla and Wild, 2012). While chronic infection with oncogenic pathogens has long been identified as a main contributor, the impact of HIV infection, particularly in the HAART era, as well as the ever-increasing incidence of type 2 diabetes represent emerging risk factors for morbidity and mortality in those regions of the world. Indeed, patients living with HIV/AIDS have an increased burden of non-communicable diseases relative to HIV-uninfected individuals, with more than 25% predicted to develop three or more non-communicable diseases by 2030 (Smit et al., 2015).

**FIGURE 1:**
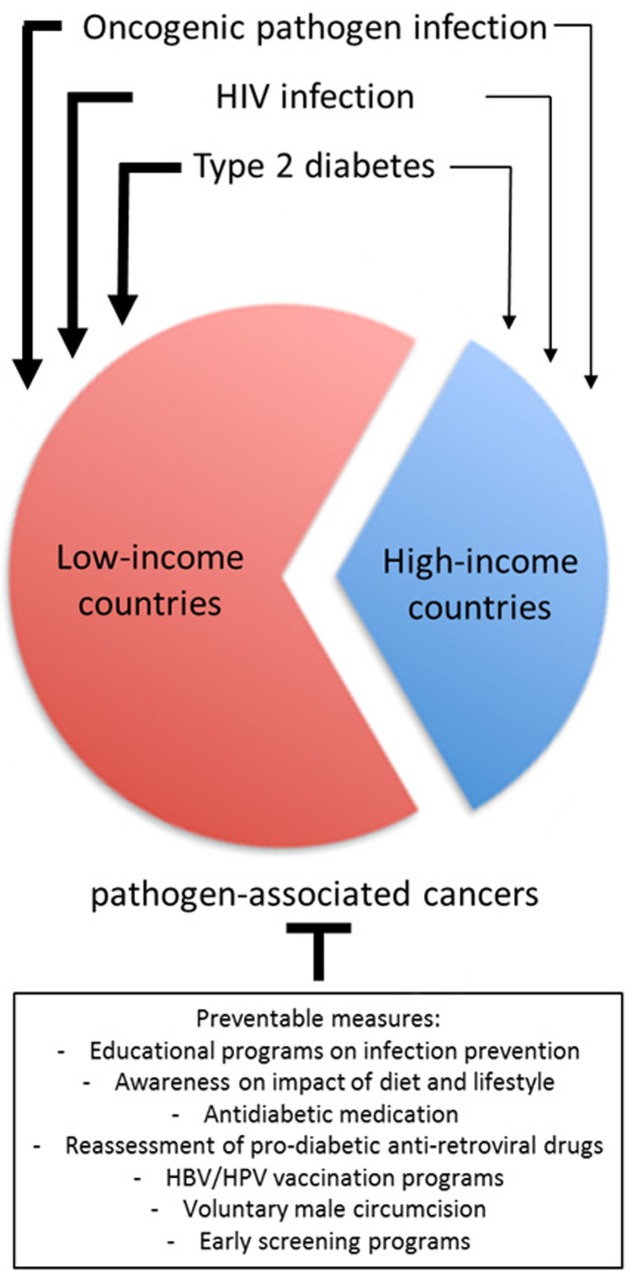
The relative contribution of oncogenic pathogen infection, HIV infection and type 2 diabetes for pathogen-associated cancers in low-income versus high-income countries and the potential prevention of these malignancies.

Globally, two-thirds of infection-attributable cancers occur in less developed countries (Plummer et al., 2016) (**Figure 1**), although the true incidence rates within the African continent are very uncertain due to underreporting and the data from GLOBOCAN often not being comprehensive. Nevertheless, low-resource settings also carry by far the largest burden of the HIV epidemic: in 2015, an estimated 36.7 million people were living with HIV globally of whom approximately 19.0 million (i.e., more than 50%) live in Southern Africa (Global AIDS, 2016). These numbers have been increasing over the years (from 31.0 million in 2002 to 36.7 million in 2016) due to the life-extending effects of HAART (Zaidi et al., 2013). Moreover, two-thirds of all diabetes cases as a consequence of rapid changes in lifestyle, urbanization and population aging are reported to occur in low- and middle-income countries (Wild et al., 2004; Hu, 2011), representing a growing public health issue in the HIV-infected population, especially as the clinical course of HIV infection in the HAART era has changed from a progressive illness with a fatal outcome to a chronic manageable disease (Deeks et al., 2013).

Both type 2 diabetes and the consequences of HIV infection share common characteristics with regard to facilitating cancerogenesis initiated by oncogenic pathogen infection, but is there a causal relationship between these two conditions? As outlined above, chronic inflammation and immunosuppression are two of the main cancer hallmarks associated with persistent oncogenic pathogen infection adverting the normal functioning of the cellular machinery. While immune dysfunction is characteristic for HIV infection, smoldering chronic inflammation is also associated with type 2 diabetes and HIV infection (particularly when treated by HAART), highlighting the importance of these two conditions as promoting environments for the development of pathogen-associated cancers. Although type 2 diabetes and HIV infection might constitute independent risk factors for pathogen-associated cancers in general, some striking synergism has been described for hepatitis virus-associated hepatocellular carcinoma. Indeed, overwhelming evidence in the literature points toward an association between co-infection of HIV and HBV/HCV together with insulin resistance and/or diabetes and a significantly enhanced risk of liver disease (Merchante et al., 2009; DallaPiazza et al., 2010; Howard et al., 2010; Salmon et al., 2012; Elkrief et al., 2014; Hadigan and Kattakuzhy, 2014; Lo Re et al., 2014; Oliver et al., 2016). This was found to be further exacerbated by certain antiretroviral drugs such as didanosine and/or stavudine (Blanco et al., 2011).

This well-documented link of HIV infection, type 2 diabetes and liver cancer is less evident for other pathogen-associated malignancies. For example, only case reports on HIV-related KS in the presence of type 2 diabetes are currently available, calling attention to the risk of delayed diagnosis of KS in patients on ART with a relatively high CD4 count (Chan and Pakianathan, 2011). Although no such case report on the link between HIV, type 2 diabetes and cervical cancer exists, an interesting recent study describes the combination of metformin, the worldwide most widely prescribed first-line therapeutic drug for type 2 diabetes and known for its antitumor properties (Kasznicki et al., 2014), together with nelfinavir, an HIV protease inhibitor. This drug combination showed promising effects on HPV-associated cervical cancer cell growth *in vitro* and *in vivo* (Xia et al., 2017). It is highly likely that other pathogen-associated cancers also benefit from the underlying cancer-friendly environment in the presence of both HIV infection and type 2 diabetes. Their cancer-promoting contributions are unlikely to be separate entities but rather present complex interactions facilitating cancerogenesis especially in a setting where these risk factors predominate.

In light of the low resources and the often deficient health care infrastructure in less developed countries, concerted efforts on cancer prevention rather than cancer treatment will likely have a sustainable effect on the reduction of cancer. The first step to prevention is the identification of risk factors and/or the etiological agent. Indeed, infections have been suggested to be one of the most important preventable causes of cancer in general, not least reflected by the significantly disparate incidence rates of pathogen-associated malignancies in industrialized versus low- and middle-income countries (**Figure 1** and Kuper et al., 2000). Highly effective prophylactic vaccines exist for some of the most common etiological agents of pathogen-associated cancers, namely HBV and HPV types 16 and 18, causing liver and cervical cancer, respectively, the two most common causes of cancer death in Africa (Sylla and Wild, 2012). Although the HBV vaccine is relatively inexpensive, national HBV infant immunization programs as seen in many African countries account only for 10% coverage in Africa (Plummer et al., 2016). The HPV vaccines are still rather expensive to produce, limiting efficient coverage of the population in the most affected areas (Hanson et al., 2015). Moreover, of the 13 HPV genotypes classified as carcinogenic (Working Group on the Evaluation of Carcinogenic Risks to Humans [IARC], 2012), the HPV vaccines currently available in low-resource settings only target the most common oncogenic genotypes 16 and 18 which account for approximately 70% of invasive cervical cancers globally. In sub-Saharan Africa however, other carcinogenic HPV types, such as HPV45 and 35, occur relatively more frequently than in other world regions as they seem to be more affected by changes in immunodeficiency levels (De Vuyst et al., 2013; Clifford et al., 2016). Therefore, even if there was saturation coverage of vaccinated populations in those regions of the world, the reduction in cervical cancer will fall well short of 100%. In this regard, voluntary medical male circumcision has a significant protective effect not only against HIV but also high-risk HPV, HBV and other sexually transmitted infections (Castellsague et al., 2002; Tobian et al., 2014; Wahome et al., 2017). Although highly cost-effective, implementation of the WHO recommended goal of 80% circumcision coverage among men aged 15–49 years in 13 countries in Eastern and Southern Africa with high HIV prevalence has been very slow and is far from being reached (Tobian et al., 2014). To reduce pathogen-associated cancers in low-resource settings both HPV/HBV vaccination and male circumcision should be advocated. Even so, efforts in the reduction of infection-related cancers are often offset by an increasing number of new cases that are associated with (among others) dietary and lifestyle factors (Bray et al., 2012). This is further exacerbated by the adverse effects of HAART on the development of type 2 diabetes and low-grade chronic inflammation seen in HIV/AIDS patients (Maartens et al., 2014). Therefore, programs to implement nation-wide prophylactic vaccination against HBV and HPV together with concerted efforts to reduce type 2 diabetes risk and the risk of acquiring sexually transmitted infections such as HIV, HBV/HCV and HPV, would have a significant impact on cancer incidence and mortality (**Figure 1**). Indeed, several randomized clinical trials have demonstrated that diet and lifestyle modifications are highly effective in preventing type 2 diabetes in different ethnic and racial groups (Knowler et al., 2002; Lindstrom et al., 2006; Ramachandran et al., 2006; Li et al., 2008).

## Conclusion

It has become evident over the last decades that the nature of comorbidities in HIV-infected individuals has changed substantially, particularly with regards to tumorigenesis and oncogenic disease progression. Although the descriptive data presented here may not lead to a definitive scientific interpretation, they clearly support the hypothesis that some of the highest cancer risk factors that predominate in resource-limited settings (such as oncogenic pathogen infection, HIV infection and type 2 diabetes) are potentially avoidable. It is suspected that there might be a link between these risk factors, particularly between HIV infection, its treatment and the onset of non-communicable diseases such as type 2 diabetes. Effective interventions including population-based vaccination against HBV and HPV together with HIV prevention and cervical cancer screening programs as well as awareness, counseling and educational programs on changes in diet and physical activity would lead to significant reductions of the cancer burden in those areas of the world. Early intervention programs not only prevent disease onset and complications but are clearly much simpler and cheaper than treating later stage disease. Research activities to understand the synergistic effects between the risk factors discussed in this review are needed and should be a focus of future scientific efforts.

## Author Contributions

GS led the conception and design of this article, drafted, revised and approved its final version. MB and SU equally contributed to drafting individual sections, while AK critically revised the article.

## Conflict of Interest Statement

The authors declare that the research was conducted in the absence of any commercial or financial relationships that could be construed as a potential conflict of interest.
